# A Conceptual Model of Long-Term Weight Loss Maintenance: The Importance of Cognitive, Empirical and Computational Approaches

**DOI:** 10.3390/ijerph18020635

**Published:** 2021-01-13

**Authors:** Darren Haywood, Blake J. Lawrence, Frank D. Baughman, Barbara A. Mullan

**Affiliations:** 1 School of Population Health, Curtin University, GPO BOX U1987, Perth 6845, Australia; darren.haywood@postgrad.curtin.edu.au (D.H.); blake.lawrence@curtin.edu.au (B.J.L.); frank.baughman@curtin.edu.au (F.D.B.); 2 WA Cancer Prevention Research Unit, School of Population Health, Curtin University, GPO BOX U1987, Perth 6845, Australia; 3 Health Psychology & Behavioural Medicine Research Group, School of Population Health, Curtin University, GPO BOX U1987, Perth 6845, Australia

**Keywords:** weight loss, maintenance, executive function, EF, social support, habit, stress, computational modeling, obesity

## Abstract

Living with obesity is related to numerous negative health outcomes, including various cancers, type II diabetes, and cardiovascular disease. Although much is known about the factors associated with obesity, and a range of weight loss interventions have been established, changing health-related behaviours to positively affect obesity outcomes has proven difficult. In this paper, we first draw together major factors that have emerged within the literature on weight loss to describe a new conceptual framework of long-term weight loss maintenance. Key to this framework is the suggestion that increased positive social support influences a reduction in psychosocial stress, and that this has the effect of promoting better executive functioning which in turn facilitates the development of healthy habits and the breaking of unhealthy habits, leading to improved ongoing maintenance of weight loss. We then outline how the use of computational approaches are an essential next step, to more rigorously test conceptual frameworks, such as the one we propose, and the benefits that a mixture of conceptual, empirical and computational approaches offer to the field of health psychology.

## 1. The Obesity Pandemic

The global prevalence of obesity has almost tripled since 1975 [1]. Recent projections suggest that almost 80% of US adults will be living with overweight or obesity by 2030, with extreme obesity (body mass index ≥ 35) becoming the most common weight class for women [2]. It is well established that living with overweight or obesity is associated with numerous negative health consequences, including increased risk of cardiovascular disease [3], type II diabetes mellitus [4], and colorectal, ovarian, renal cell, postmenopausal breast, gallbladder and thyroid cancers [5]. In addition, many individuals living with overweight or obesity experience negative psychosocial issues, including increased risk of depression [6], anxiety [7], and loneliness [8], as well as a long-term increased risk of dementia in later life [9]. It is therefore increasingly clear that living with overweight or obesity has a detrimental impact on an individual’s physical and mental health and more research is needed to address this growing global health crisis.

Over the past 30 years, people living with overweight or obesity have been encouraged to increase physical activity and adopt healthier eating patterns to limit or reduce weight gain. However, the increase in the obesity epidemic (now known as the obesity pandemic, [10]) suggests that conventional strategies for prevention (i.e., diet and exercise interventions) have not been effective at preventing or reducing obesity [11]. Evidence from a recent systematic review and network meta-analysis of 121 randomised controlled trials of 14 popular diets (e.g., Atkins, Weight Watchers) showed that while almost all diets resulted in short-term (i.e., 6 month) weight loss and improved blood pressure, these beneficial effects largely disappeared at long-term (i.e., 12 month) follow up [12]. Similar results have been found in physical activity interventions for weight loss. A systematic review by Dombrowski and colleagues [13] found that among randomised controlled trials of physical activity interventions, participants with obesity only maintained approximately 1.50 kg of weight loss at 12-month follow up assessments. To experience significant health benefits and dramatically reduce health risks associated with obesity, many people living with this extreme degree of weight gain need to reduce their body weight by 30% to 40%. This often equates to 30 to 60 kg of weight loss that needs to be maintained long-term. However, existing evidence shows that most popular diets do not lead to long-term weight loss and most physical activity interventions lead to an inconsequential proportion of weight loss for people with obesity. For most people, obesity develops over many years (or decades) and is likely associated with underlying psychological trauma or cognitive deficits that limit the therapeutic potential of conventional strategies (i.e., diet and exercise [14]. Meta-analysis shows that many people with overweight or obesity demonstrate deficits when performing tasks that involve high-order cognitive processes [15,16], which may help explain why long-term weight loss maintenance appears to be an insurmountable challenge for most people living with obesity.

In this paper we describe the current theoretical and empirical literature linking executive functioning to this global pandemic, and the mixed findings relating to changes in executive functioning abilities during weight loss. We follow this by proposing a preliminary framework of successful long-term weight loss maintenance that encompasses the interactive effects of executive functioning, social support, psychosocial stress and habit. We end by outlining an approach to test and extend this framework and demonstrate the utility of computational modelling in health psychology.

## 2. Executive Functioning and Overweight and Obesity

A number of higher-order cognitive processes (e.g., attention, problem solving, and planning) underlie human cognition [17,18,19] and there is consensus that these processes are regulated and controlled by a collection of executive functions, that coordinate and organize functioning of the cognitive system, thereby governing goal-directed behaviour [17,20,21]. Executive functioning is important to consider with regards to overweight and obesity as executive functions coordinate and organise our higher-order cognitive processes to ensure successful execution of goal-oriented activities. It is therefore understandable that executive function deficits may be associated with behaviours that impact our health. Following Miyake et al. [21] seminal work, agreement has emerged that executive functioning consists of three separate components that operate in concert: updating, shifting and inhibition. Updating monitors the contents of the working memory; manipulating and replacing the contents to ensure the most relevant information is available for goal attainment. Shifting controls the engagement and disengagement of attention to better facilitate the fulfilment of the current goal. Inhibition is the ability to suppress the initiation of a predominant or automated response that does not best facilitate the attainment of an immediate goal [21].

Evidence shows that impaired executive functions are related to a range of negative health behaviours including smoking (e.g., [22]), binge drinking (e.g., [23]), risky sexual behaviours (e.g., [24]), and poor sleep hygiene [25]. More recently, meta-analytic evidence shows that people living with overweight or obesity also experience executive function deficits [15,16]. Furthermore, research shows that executive functioning may have a significant influence upon whether an individual living with overweight or obesity can successfully lose weight (short-term) and maintain weight loss (long-term) (see [26]). The functional association between executive functioning and weight loss maintenance is of particular interest due to the general ineffectiveness of weight loss interventions in the long-term [12,13].

Theories and explorations regarding the association between executive functioning and overweight and obesity can be used to explain (a) how deficits in executive functioning might be related to overweight and obesity, and (b) how a high ability of, or improvement to, executive functioning can positively influence weight loss, and the maintenance of weight loss long-term. Current theories of proposed mediators between executive function and overweight and obesity center around components such as habit, dietary restraint, goal setting, self-regulation and action planning (e.g., [26,27,28]). For example, executive function may impact the development and performance of healthy dietary habits [29], as well as act to inhibit the enactment of unhealthy habitual behaviours [27]. Mediators, such as habit, that link executive functioning to overweight and obesity are most often suggested to affect both physical activity participation and dietary intake, thereby influencing an individual’s caloric input and output. The difference between an individual’s caloric input and output over-time then influences (a) weight gain, and (b) the success of weight loss attempts.

Gettens and Gorin [26] propose a model of weight loss and long-term maintenance within which the executive function components of updating, shifting and inhibition each affect a specific set of health behaviour-related mediators which in turn influence one’s ability to undergo successful initial weight loss and/or long-term weight loss maintenance. For example, the increased ability to update working memory facilitates better self-monitoring. The ability to shift mental sets positively affects an individual’s ability to exercise flexible dietary restraint. One’s ability to inhibit facilitates automatic positive health behaviours. In combination, each of these mediators are held to facilitate long-term weight loss maintenance [26].

## 3. Can Training Executive Functions Improve Weight Loss Maintenance?

A range of cognitive remediation programmes have been developed in an attempt to improve executive functioning in people with overweight and obesity (e.g., [27,30,31,32]). These programmes attempt to enhance individuals’ executive functioning with the goal of improving their health behaviours [27,30,31,32]. Typically, these programmes either train executive functioning through a programme of repeated completion of cognitive tasks or involve one-on-one practitioner-led exercises aimed to promote thought reflection and improve cognitive strategies [27,30,31,32]. A proportion of these programmes have demonstrated a degree of weight loss and weight loss maintenance in participants over the short term (e.g., extending to 3 months, [31]). However, the mechanisms in which these interventions might improve executive function and weight loss/weight loss maintenance remain unclear, and the efficacy of cognitive remediation therapy for long-term weight loss maintenance remains unclear. Furthermore, the majority of cognitive remediation interventions to date integrate or precede cognitive training with other interventions, such as cognitive behavioural therapy [30], or behavioural weight loss group programmes [27,31,32]. These confounding variables may limit our knowledge of the specific efficacy and generalisability of cognitive remediation for weight loss and weight loss maintenance. For example, it is possible that the cognitive behavioural therapy would facilitate psychosocial stress reduction and this stress reduction, rather than the cognitive remediation therapy, is primarily responsible for weight loss success. Lastly, considering the prevalence of overweight and obesity worldwide, for cognitive remediation therapy to be impactful on a large scale its feasibility and acceptability in general populations with overweight and obesity needs to be demonstrated. Although more work needs to be done to demonstrate the efficacy of cognitive remediation for weight loss and long-term weight loss maintenance as a large-scale public health measure, cognitive training demonstrates, at least in the short term, that executive functions are malleable.

Evidence shows that executive functioning can change over time, and multiple psychosocial factors may affect executive functioning ability. For example, stress [33,34], depression [35], and sleep disturbance [36] negatively impact executive functioning. Given the importance of executive functioning abilities to long-term weight loss and maintenance, it is important to understand how executive functioning ability may change over time, and what might negatively impact executive performance for people with overweight or obesity.

## 4. The Impacts of Weight Loss on Executive Functioning

The paradox of executive function performance and long-term weight loss maintenance suggests that for some people, weight loss may negatively impact executive functioning (despite improved metabolic health associated with weight loss), which potentially limits the cognitive capacity of an individual to successfully pursue weight loss goals over time. Gettens and Gorin [26] note the mixed findings associating weight loss to changes in executive functioning. Some studies show executive function declines in people undergoing a period of weight loss [37,38,39], whereas other studies show improvements in executive function during weight loss for people with overweight and obesity [39,40], and further research reports no changes in executive function during weight loss interventions [41,42,43,44]). Further demonstrating this heterogeneity, in Sievo et al. [45] meta-analysis and systematic review, five of the eight studies assessing the impacts on executive functioning of intentional weight loss found intentional weight loss to be associated with better executive performance [39,40,43,46,47], and three of the eight studies found it to be associated with worse executive performance in some domains [37,40,46].

A better understanding of what contributes to this high degree of heterogeneity would therefore help explain why some individuals are more successful than others in long-term weight loss and weight loss maintenance. If an individual experiences executive function decline during a period of weight loss, they may be more prone to performing negative health behaviours thereby negatively influencing their success. Conversely, an increase in an individual’s executive functioning during a weight loss period may facilitate action planning, dietary restraint, stress regulation and the development of healthy habits, that may facilitate long-term weight maintenance (see [26]). While a thorough understanding of the longitudinal effects of weight loss on executive functioning are lacking, several explanations have been given for why executive functioning may decline during weight loss for people with overweight and obesity. For example, weight loss efforts (e.g., dietary restraint), and psychological factors often present during weight loss, such as a preoccupation with weight loss and one’s body image, are said to place a burden on an individual’s cognitive resources [41]. This lack of available cognitive resources may then negatively impact executive functioning [26,41]. This mirrors evidence showing high levels of psychosocial stress during weight loss may negatively impact executive functioning [33,34], once again possibly due to its impact on cognitive resources. It is therefore important to understand how to best support individuals with overweight and obesity during weight loss attempts to minimise their experience of factors that may negatively affect their executive functioning, and thereby their long-term weight loss and weight loss maintenance efforts.

## 5. Social Support and Weight Loss Maintenance

Perceived social support that potentially improves adherence to long-term weight loss involves a mutual understanding and collection of expectations, as well as empathy and trust [48]. Social support consists of two components: structural support; which is the general availability of those that can provide support, and functional support; which is perceived by the individual [49]. Cohen [50] explains that social networks provide an individual with emotional, material, and informational resources which aid in psychosocial stress management. The importance of stress management during attempted weight loss and maintenance is demonstrated throughout the literature (e.g., [51,52]). A particularly important consideration with regards to weight loss maintenance is the interaction between psychosocial stress levels and executive functioning.

### 5.1. Psychosocial Stress, Executive Function and Social Support

**Figure 1:**
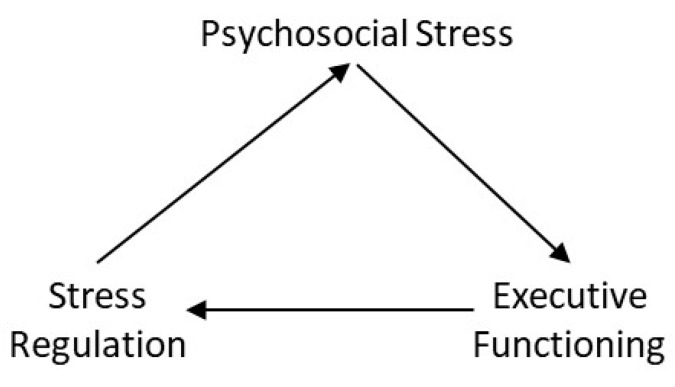
A cyclical model of psychosocial stress, executive functioning and stress regulation.

There may be a bidirectional association between stress and executive function. For example, high executive function ability is associated with successful regulation of psychosocial stress [53], and high levels of psychosocial stress may impair executive function [33,34]. We suggest psychosocial stress is indirectly related to difficulties in maintaining weight loss through (a) its tendency to negatively impair executive functioning, leading to poor weight loss maintenance behaviours and (b) difficulties in regulating current or future psychosocial stress. A cyclic association may therefore reciprocate impaired executive functioning and high levels of psychosocial stress. The cyclic association between psychosocial stress, executive functioning and stress regulation is shown in Figure 1.

Social support has been identified as a contributing factor that differentiates between people who do and do not maintain weight loss over time [52]. People are at greater risk of weight regain after weight loss if they experience social stress or stressful life events (e.g., [54]) and research points toward social support as an important component to successful weight loss maintenance [55,56,57], likely primarily due to its role in psychosocial stress regulation [50]. Individuals who participate in weight loss maintenance social groups, either online or face-to-face, consisting of their peers, typically have high levels of weight loss maintenance success [54,58]. Furthermore, evidence shows that interventions of social support from friends lead to better weight loss and weight loss maintenance outcomes (e.g., [59]). However, other research has found evidence to the contrary. For example, correlational work has found low levels of perceived family encouragement to be associated with more success in long-term weight loss maintenance, and similarly, other research has found encouragement from friends to be associated with weight regain [60]. In order to shed light on these mixed findings Karfopoulou et al. [61] explored social supports’ association with weight loss maintenance. Karfopoulou et al. [61] compared the social support of 122 individuals who intentionally lost and then regained 10% or more of their body weight with those who lost and maintained 10% or more of their weight over one year. Contrary to expectations, participants who regained their weight loss received more support than those who maintained their weight loss [61]. However, those who maintained their weight loss reported receiving social support that was active in nature, such as their family participating in their positive health habits, and people giving them compliments for their participation in healthy behaviours [61]. Whereas, those that regained their weight received less active participation and compliments from support givers and more support through instructions and encouragement of healthy lifestyle behaviours [61]. Kafopoulou et al. [61] suggest that positive social support facilitates long-term weight loss maintenance, while instructive social support may negatively impact weight loss maintenance attempts.

Green et al. [46] explored the differences in weight loss, stress, and executive functioning in two groups with or without weight loss support. The supported group participated in a short-term (8-week) weight loss programme comprising a structured calorie restricted diet, weekly weigh-ins and participation in a weekly group support session. The unsupported group was instructed to follow any diet plan for the 8 weeks as long as it did not involve membership of an organised group. At postintervention both groups had lost a comparable amount of weight and executive functioning did not significantly differ between the groups [46]. However, following baseline testing, unsupported dieters saw a significant increase in cortisol levels (related to stress) and a subsequent decrease in executive functioning abilities in the first week of the programme. This may suggest that during stressful periods of weight loss, such as starting a weight loss diet, social support may positively impact stress and executive functioning. Due to the short-term nature of this research the authors were unable to conclude how this may impact long-term weight loss and weight loss maintenance. However, it is possible that over time the participants would have been subject to further psychosocial stress resulting from weight loss/daily life factors and events. Social support may therefore become increasingly important to weight loss maintenance over time, due to cumulative stress minimisation and regulation protecting executive functioning ability. The current collection of empirical findings and theoretical accounts, in addition to preliminary evidence showing that a combination of high executive functioning and high levels of social support results in improved long-term weight loss maintenance [62], points toward the importance of this relationship. However, further research needs to directly explore the interactions between social support, psychosocial stress, executive function and long-term weight loss maintenance. A further consideration is that improved psychosocial stress regulation from positive social support [62] may positively impact executive functioning and facilitate the function of other important weight loss maintenance mechanisms, for example, habits.

### 5.2. Executive Functioning and Habit

Habits are highly learned processes that produce autonomous behaviours in response to cues [63]. Habits have been the subject of extensive research with regards to health behaviours. Both unhealthy and healthy habits have been used to predict a variety of health behaviours relevant to weight loss maintenance. For example, habit is associated with positive health behaviours such as fruit and vegetable consumption [64] and physical activity [65], as well as negative health behaviours such as unhealthy eating and sedentary behaviours [27]. The dual process theory of self-regulation [66] proposes bottom-up automatic processes, such as habits, can be controlled and regulated by higher-order cognitive processes, such as executive functioning, to direct behaviour toward goal attainment [67]. The dual process theory also suggests that executive functions may play a vital role in the regulation of unhealthy habits [27]. For example, Allom et al. [27] found that improvements in executive functioning were associated with the reduction of both sedentary behaviour, and unhealthy eating habits.

Although understudied in the health literature, executive functioning may also facilitate the development and execution of healthy habits. Executive functioning is said to govern goal-directed behaviour [17,20,21], for example, a person wanting to include vegetables in every meal they prepare. Cooper et al. [68] describes that with consistent performance of the behaviour it may move from a nonroutine system to a routine system represented as a collection of sequential routine actions (i.e., a habit), over time. The development of a habit requires the consistent performance of the behaviour in response to a cue [69]. Regardless of the cue, in the early stages while the behaviour (i.e., including vegetables with each meal) is governed by the nonroutine system, executive functioning is required to regulate the behaviour toward attaining that goal [17,18,19,20,21,68,70]. Therefore, in order for habit development and subsequent execution, executive functioning is required.

The importance of social support with regard to habit relates to psychosocial stress and its impacts on executive functioning. As previously demonstrated positive social support, defined as receiving social support that is active in nature rather than instructive [61], has the potential to mitigate psychosocial stress during weight loss and weight loss maintenance. Low levels of positive social support have the potential to result in higher levels of psychosocial stress, which can negatively affect executive function during weight loss and maintenance. Lower levels of executive function may result in a poorer ability to inhibit and break unhealthy habits [27], as well as negatively influence the consistent performance of goal-based positive health behaviours, which in turn, may reduce the movement of the positive behaviour from the nonroutine to the routine system [68]. Therefore, high levels of positive social support may facilitate long-term weight loss maintenance through facilitating the internal environment necessary for successful healthy habit development and unhealthy habit breaking.

## 6. A Conceptual Framework of Long-Term Weight Loss Maintenance: Executive Functioning, Social Support, Psychosocial Stress and Habit

**Figure 2:**
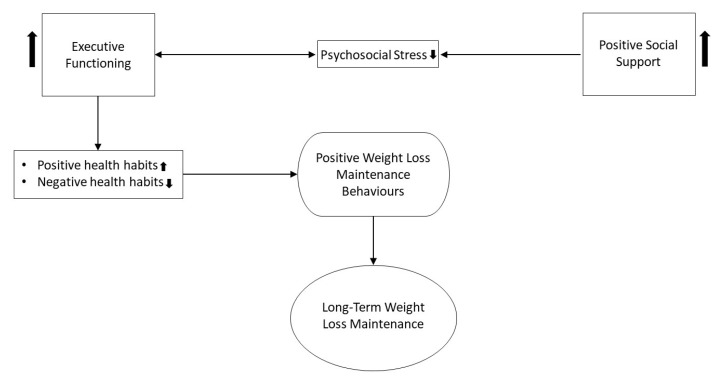
A conceptual framework of long-term weight loss maintenance: executive functioning, social support, psychosocial stress and habit.

Overall, positive social support is a promising, assessable component that has the potential to significantly improve an individual’s long-term weight loss maintenance success. However, it has been unclear through which mechanisms positive social support may positively influence weight loss maintenance. Here, we extend on the work of Karfopoulou et al. [61] and Gettens and Gorin [26], by providing a preliminary model of weight loss maintenance incorporating executive function, social support, psychosocial stress and habit contributing either directly, or indirectly to positive weight loss maintenance behaviours (shown in Figure 2). It is possible that aspects of the model, such as positive social support, have varied dynamics within a younger demographic due to social environment differences (e.g., parent and schooling dynamics). Therefore, like Gettens and Gorin [26], this is a framework intended as a model to elucidate potential mechanisms of adult weight loss maintenance. This is an initial model to be tested, extended and revised. For example, positive social support may occur through multiple interconnected social relationships including family, friends and acquaintances which together may have dynamic effects on an individual’s social support experience. Here, we incorporate social support at the macrolevel and future research may look to make these interconnections explicit in the model. Future versions of the framework may also incorporate other psychosocial and cognitive components, as well as biological elements, and each executive functioning component individually. Lastly, extensions of the model might also include specific positive weight loss maintenance behaviours and importantly, the functional mechanisms of each component must be unpacked and tested. Summarising the sections above, Figure 2 displays our conceptual framework.

While we believe the conceptual model described here offers a potentially important framework for better understanding long-term maintenance of health-related behaviours in weight loss, the true relation between factors is unspecified. In this section, we outline what is required to move from a verbal theory that describes a portion of the phenomenon of interest, to a fuller theory that explains how and why some individuals are successful (and others are not) in their effort to maintain long-term weight loss.

## 7. Framework Validation and Extension

Scientific models are simplified representations of a target object or phenomenon. They are referred to as “simplified representations” because the process of developing a model requires the extraction of a limited set of key variables. The objective in using models is to reveal the causes of differences in outcomes related to the object or phenomenon of interest. In the paper we propose a conceptual framework, in the form of a verbal model, that we believe provides a good representation of a system of interrelated components that helps explain long-term weight loss maintenance success. However, it is important we are able to explicitly test this representation. In a verbal model, such as our conceptual framework, it is easy to mask discrepancies between the theorised role of a factor or variable and associations between measures of that variable and the outcome. In a computational model, this is not the case. Either a model succeeds in simulating the target phenomenon or it fails. Crucially, the reasons for it failing are just as important as the reasons for it succeeding. For if a model fails, it is either because the (A) model is wrong (wrong decision to steps/questions above), or (B) the theory is wrong. Either of these outcomes are informative and help to progress our understanding of the nature of the phenomenon of interest, in this case long-term weight loss maintenance. We suggest that the validation and extension of our model of long-term weight loss maintenance should undergo traditional empirical (e.g., cross-sectional and longitudinal) and computational assessment. In the following sections, we provide context of model validation within a computational approach by outlining verbal theory approaches and the utility of computational modelling in health psychology using our conceptual framework as an example. We then provide an example of a well-known computational model to elucidate computational approaches.

### 7.1. Verbal Models—What Are They Good For?

Within psychology it is common to find verbal models (sometimes referred to as conceptual models/frameworks) describing the component parts of theorised interactions between core variables relating to psychological phenomena. For instance, one can find models describing the theorised relations underlying personality, visual-spatial memory, speech disorders, health beliefs, infant attachment, word recognition, memory, attention and more. Often these descriptions are accompanied by visual models—box-and-arrow diagrams representing those conceptual relations.

A large part of the appeal of verbal models has been the fact that they make intuitive sense, by providing a kind of structure to a problem. However, as we show in this section, on their own, verbal models fail to provide adequate means for progressing a proper understanding of psychological issues. By contrast, computational models provide explicit frameworks to test the role of theorised variables, and thus offer a way of fully evaluating explanations of psychological phenomenon. Next, we detail how this is achieved, and thus how computational models offer an advantage over verbal models.

As we have stated, our conceptual framework of long-term weight loss maintenance is not intended to represent all variables relating to the phenomenon. However, our framework helps reveal the central problem with verbal models. This problem is that whilst each of the variables are evidenced to have some relation, either directly or indirectly, to long-term weight loss maintenance, their precise contributions are difficult to determine. Specifically, holding all other variables constant, it would be difficult to determine exactly how, for example, the increase of habit strength by one unit for a certain healthy behaviour would affect the outcome of weight loss maintenance over time, or precisely what changes would occur in long-term weight loss maintenance if, for a given level of psychosocial stress, a specific unhealthy habit was adopted.

These issues illustrate the central flaw of verbal models, and are pertinent to any existing model from within the discipline of health psychology, where one can find a heavy reliance on verbal models. As with our model of long-term weight loss maintenance, none of these other verbal models permit a full evaluation of specific assumptions relating to theorised variables. We believe the time has come for health behaviour psychologists to move away from just verbal models, to approaches that allow the putative role of theorised variables to be more fully evaluated. For this, computational models are aptly suited.

### 7.2. The Role of Computational Models in Understanding Behaviour

Examples of the application of computational models are plentiful. One can find computational modelling used extensively in the study of complex systems within the formal sciences (disciplines that include, e.g., mathematics, systems and decision theory), e.g., [71], natural sciences (e.g., physics, chemistry, and biological systems), e.g., [72], and applied and social sciences (e.g., epidemiology, economics, and psychology), e.g., [73]. For instance, epidemiologists use computational models to simulate the rate of transmission of viruses, economists use models to predict future iron ore markets, meteorologists model long-range climate change patterns, and cognitive scientists use models to study such things as problem-solving and decision-making in humans.

Whilst computational modelling refers variously to a range of approaches which can be differentiated broadly according to their type (e.g., neural networks, dynamical systems, symbolic models and hybrid systems), the approaches are unified by several fundamental requirements (see, e.g., [74]). Specifically, during the development of a model, the modeller is forced to explicitly answer a number of central questions, such as:

(1) What are the essential or “key variables” of the model (and, thus what is extraneous)?
(2) How should the key variables be instantiated and what is the range of properties or values that should be given to each variable?
(3) How should the relation between key variables be expressed?
(4) What criteria should be used to evaluate the model’s success in simulating the phenomenon?

How those questions are handled, ultimately determines how well a model simulates a target phenomenon, and models can fail when the wrong decisions are made with respect to any one of the questions above. So, whilst challenging, it is this requirement to provide unambiguous answers in a computational model that makes computational approaches so valuable, and which distinguishes them from verbal models.

Although the principles, and broadly described benefits of computational modelling may be straightforward, models themselves can remain slightly mysterious for some. In an effort to demystify, and thus make computational models more tangible, we next set out the details relating one of the most well-studied models originating from the study of ecosystems—The Lotka−Volterra, or predator−prey model.

### 7.3. The Role of Computational Models in Understanding Behaviour

The Lotka−Volterra model see, e.g., [75,76] captures the complex dynamics of change within two interdependent populations (for simplicity, we refer to these populations of predators and prey, as foxes and rabbits, respectively).

**Figure 3:**
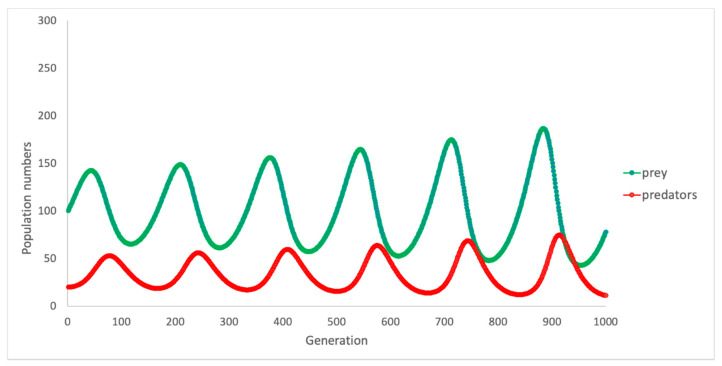
Change in predator and prey numbers over time: base model.

In nature, where these populations exist, one can expect that if resources are plentiful for the rabbits (i.e., there is an abundance of food) and when there are few threats (e.g., few foxes), then the population of rabbits will grow. However, as the number of rabbits grows, their greater number also provides greater opportunity for the foxes that prey on them. Thus, the fox population will grow, and rabbit numbers will fall. However, the downturn in the population of rabbits, and increasing competition amongst the fox population for fewer resources (i.e., rabbits) leads to a natural decline in the numbers of foxes. Figure 3 depicts the observed patterns of change in these populations over time, via two oscillating “growth curves” representing foxes (red line) and rabbits (green line).

The Lotka−Volterra Equation models this pattern of non-linear and dynamic change via coupled differential equations. The model specifies precisely how change in one population effects change in the other. Equation (1) below shows one form of the Lotka−Volterra model. The Equation shows the key variables that have been used to model the phenomenon of change observed in these populations. The size of two populations is specified by *x* (the prey; in this case rabbits) and *y* (the predators; in this case foxes). The population of the rabbits grows at an exponential rate *a*, and is constrained by resources in the environment *K* (referred to as the carrying capacity) and the frequency with which the rabbits come into contact with and are killed by the foxes *Myx*. In the lower part of the Equation, the population of foxes, *y*, grows at rate *b* and is constrained by the resources available (i.e., rabbits) and the effectiveness of their predation on rabbits *Mbx* (i.e., the proportion of times in which an encounter with a rabbit results in dinner for the fox).

(1) $$Prey:\;\;\;\frac{dx}{dt}=ax\;\left(\frac{1-x}{K}\right)-Myx$$

(2) $$Predator:\;\;\;\frac{dy}{dt}=\left(Mbx-c\right)\;y$$

Equation (1). The Lotka−Volterra Model.

The model fully determines the interactions between variables within each population and the interaction between populations. With this model fully specified we can now take any variable (or combination of variables) and evaluate the precise consequences of altering their values.

**Figure 4:**
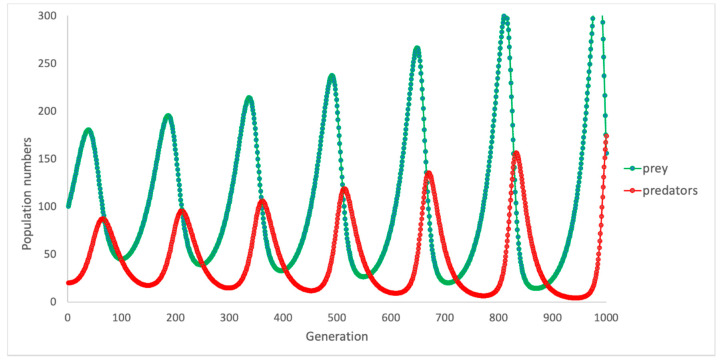
Change in predator and prey numbers over time: base model: increased prey growth.

For instance, if we wanted to ascertain precisely what effects we could expect to see for a different growth rate in the rabbit population (e.g., a different species was more productive) we could isolate that specific part and see what outcomes result. In Figure 4 below, the parameter in the Equation determining rate of growth has been increased, and one can see the differences compared to the earlier Figure 3.

**Figure 5:**
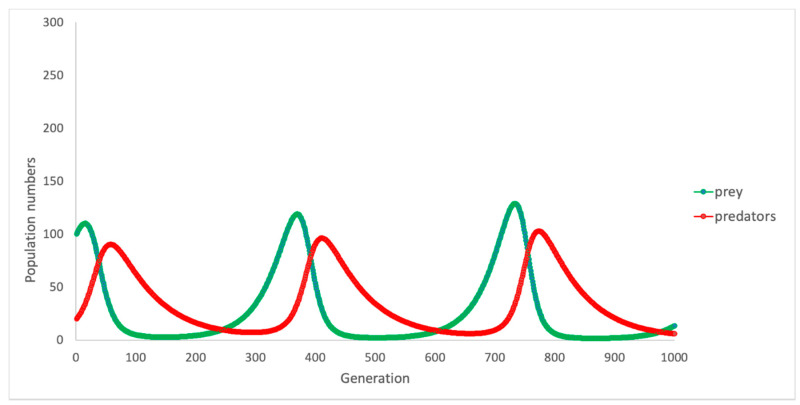
Change in predator and prey numbers over time: base model: increased rate of attack.

Alternatively, if we were interested in knowing what effects would occur if the rate of mortality were different, we could also isolate the part of the Equation *Myx*, which determines the rate of attack on rabbits. Increasing this parameter also affects both populations in drastic ways. Figure 5 shows an increased rate of attack and the net effect on rabbits and foxes. The impact of changing this rate has the effect of almost wiping out both populations for several generations.

The variables instantiated within this version of the Lotka−Volterra model are by no means the only variables concerned in accounting for the dynamics of population change. Again, certain simplifications have been made in this model to allow the main points to shine. For instance, in the form expressed in Equation (1), the model assumes that foxes are the only predator, and that nothing else affects the numbers in these populations (e.g., there are no other predators, and no disease, injury, etc.). Clearly, this is not realistic. However, this was not the point of using this model (indeed, any of these additional variables may be included if desired). The point of using the Lotka−Volterra model is that even in the more basic form it accounts for the natural phenomena that exist in complex and dynamic systems. This is important, as it shows that, as with the kinds of issues that are important to the study of health psychology, it is the extraction of key variables that ultimately determines whether real progress is made towards understanding phenomena like weight loss.

By contrasting verbal and computational models we aimed to show that in using computational models, theorised variables can be unambiguously set out and the effects of differences in the properties of those variables can be empirically assessed—this process lays open to direct scrutiny all aspects of the model in a way that verbal models cannot. To make headway in understanding how to help individuals maintain weight loss long-term, we need better theories—and to achieve better theories we need to move away from just verbal models, and finally begin testing explicit conceptions of the role variables may play in affecting those outcomes.

Our conceptual framework of long-term weight loss maintenance should be tested and extended via computational modelling. Computational modelling would allow for the mechanisms underlying each theorised variable to be made explicit and its various potential effects assessed. For example, it would be possible to examine the precise contributions and interactive effects of each executive function on habit formation within a functional system consisting of the variables within our framework. This would result in a more detailed framework where we “open” and examine each box in the box-and-arrow model.

## 8. Conclusions

In this paper we present the case for a conceptual framework of long-term weight loss maintenance with these interacting components: social support, psychosocial stress, executive functioning, health behaviour habits, and positive weight loss maintenance behaviours. Ultimately, we claim that positive social support is functionally associated with psychosocial stress levels. Further psychosocial stress is functionally associated with executive function and thereby indirectly associated with the development of positive health habits, and the breaking of negative health habits. The respective development and breaking of these habits then heavily contribute towards an individual’s overall long-term weight loss maintenance-facilitating behaviours, finally resulting in their net ability to maintain weight loss over time. We then suggested that the validation of this model of weight loss maintenance should undergo not only traditional empirical assessment (e.g., cross-sectional and longitudinal) but also computational assessment. We ended the paper by providing commentary on the utility of computational models in health psychology and provide a working example of a computational model.

## Data Availability

Not applicable.
